# Factors Associated With the Intention to Use mHealth Among Thai Middle-Aged Adults and Older Adults: Cross-Sectional Study

**DOI:** 10.2196/63607

**Published:** 2025-03-07

**Authors:** Nida Buawangpong, Wachiranun Sirikul, Penprapa Siviroj

**Affiliations:** 1 Department of Family Medicine, Faculty of Medicine, Chiang Mai University, Chiang Mai, Thailand; 2 Department of Community Medicine, Faculty of Medicine, Chiang Mai University, 110, Intrawarorot road, Sriphum, Meaung, Chiang Mai, 50200, Thailand, 66 53935472; 3 Center of Data Analytics and Knowledge Synthesis for Health Care, Chiang Mai University, Chiang Mai, Thailand; 4 Environmental and Occupational Medicine Excellence Center, Faculty of Medicine, Chiang Mai University, Chiang Mai, Thailand; 5 Department of Biomedical Informatics and Clinical Epidemiology, Faculty of Medicine, Chiang Mai University, Chiang Mai, Thailand

**Keywords:** mHealth, mobile healthcare, older adults, elderly, aging, questionnaire, smartphone, mHealth usage, intention to use

## Abstract

**Background:**

Mobile health care (mHealth) apps are emerging worldwide as a vital component of internet health care, but there are issues, especially among older adults.

**Objective:**

We aim to investigate the factors influencing the intention to use (ITU) mHealth apps, focusing on those with and without prior mHealth experience.

**Methods:**

A cross-sectional study conducted from August 2022 to July 2023 included Thai citizens aged 45 years or older. Self-reported questionnaires collected data on sociodemographic information, health conditions, smartphone or tablet ownership, and mHealth usage experience. The Thai mHealth Senior Technology Acceptance Model questionnaires with a 10-point Likert scale evaluated mHealth acceptance. A multivariable logistic regression analysis, adjusted for age, gender, education, income, and living area, was performed for 2 subgroups: those who used ITU mHealth apps and those who did not.

**Results:**

Of 1100 participants, 537 (48.8%) intended to use mHealth apps, while 563 (51.2%) did not. The ITU group had a younger average age, higher education levels, higher income, and fewer underlying diseases compared to those who did not intend to use mHealth apps. For those who had never used mHealth apps, having a smartphone was strongly associated with higher odds of ITU (adjusted odds ratio 2.81, 95% CI 1.6 to 4.93; *P*<.001), while having any underlying disease was associated with lower odds of ITU (adjusted odds ratio 0.63, 95% CI 0.42 to 0.97; *P*=.034). Higher acceptance levels, characterized by a positive attitude toward mHealth and lower fear of making mistakes, were also associated with higher ITU. For those with prior mHealth experience, acceptance in areas such as perceived ease of use, gerontechnology anxiety, and facilitating conditions was significantly associated with ITU.

**Conclusions:**

Among inexperienced users, a positive attitude toward mHealth significantly enhanced ITU. Conversely, having an underlying disease decreased ITU, indicating a need for tailored mHealth apps. For experienced users, acceptance levels in areas such as ease of use and gerontechnology anxiety were crucial. Future research should explore specific mHealth apps for more targeted insights.

## Introduction

Mobile health care (mHealth) apps, a vital component of internet health care, are emerging worldwide. These apps have attracted a wide range of health care services and are proven effective in solving health problems. The functions of mHealth are diverse, including patient monitoring, diagnosis, personal care, psychological health, educational apps, and social networking [1]. There is growing evidence of the health benefits of mHealth. Mobile devices, with their integrated sensors and features, help health care professionals treat patients with continuous connectivity. These apps are useful for collecting data related to physical activity, human body images, and other health care aspects [2,3]. For example, using mHealth in chronic disease management has shown improvements in symptoms and reduced hospitalizations for patients with asthma, chronic obstructive pulmonary disease, heart failure, and diabetes [4].

However, there are several issues with current mHealth apps, particularly among older adults. Studies show that only 60% of older people intend to use mHealth [5]. Older adults may have physiological changes according to aging such as visual and hearing decline. They may also be unfamiliar with technology and face difficulty learning new skills [6]. Some users experience technical problems with their smartphones when using these apps. Additionally, public awareness of mHealth apps is low, and their usability is not as good as expected [7].

Understanding the factors that affect the intention to use (ITU) mHealth among people with and without prior experience is crucial for enhancing its future usage [8]. Therefore, the primary objective of our study was to investigate the factors associated with ITU, focusing on 2 specific subgroups: those with prior experience using mHealth apps and those without. This study also examined information regarding device ownership, experience with mobile apps and mHealth, and technology acceptance. These findings aimed to gain a better understanding of mHealth usage and identify potential opportunities for implementing mHealth in a community-dwelling population, especially among older adults.

## Methods

### Study Design

We conducted the cross-sectional study from August 2022 to July 2023 using a nationwide web-based survey and a community survey. The online survey was disseminated through various social media platforms, such as department websites, Facebook, Line, Twitter, and Instagram. The investigator teams, which included medical students and health care personnels from primary care units across 10 subdistricts in Chiang Mai province, distributed the community survey. The respondents to both the online and community surveys used the REDCap (Research Electronic Data Capture; Vanderbilt University) survey platform to self-complete the questionnaires. The STROBE (Strengthening the Reporting of Observational Studies in Epidemiology) statement guided this study’s reporting [9].

### Ethical Considerations

The institutional review board of the Faculty of Medicine, Chiang Mai University approved this study’s ethical consideration (COM-2565‐09079). Before participating in this survey, all respondents provided their informed consent in accordance with the screening questionnaire and study information page. Participants were compensated with an incentive of 100 Thai Baht (3 USD) for completing the questionnaires. No identification data were recorded, and respondents were permitted to remain anonymous during the online survey. The community survey used the identification data of eligible participants exclusively for recruitment purposes within each target area. These data were not documented in either the survey form or the study database.

### Participants

Study participants were middle-aged Thai citizens aged 45‐60 years or older at the time of the survey [10,11]. Our study’s inclusion criteria required participants to be able to read and communicate in Thai and to have no underlying conditions or diseases that would hinder their ability to complete the survey or use mHealth apps (eg, dementia, active psychological problems, or severe visual impairments). This study excluded respondents who did not complete the survey, spent less than 2 minutes on it, or spent more than 60 minutes on it.

### Data Collection

#### Participant Characteristics

We used self-reported questionnaires to collect data on participant characteristics, including sociodemographic data, underlying health conditions, owning a smartphone or tablet, and experience using the devices and mHealth apps. The initial section of the questionnaires included information regarding mHealth to ensure that the participants comprehended its definition, related concepts, and intended use.

The sociodemographic data included age, gender, marital status (single, married, and separate, divorced, or widowed), living status (alone, with family, and with others), living areas (urban, suburban, and rural), education levels (no education, primary, secondary, high school, vocational training, preuniversity, bachelor’s degree, and master’s degree), and income per month (<10,000 in Thai Baht, US $ 274; 10,001‐30,000 in Thai Baht, US $275-$819; and >30,001 in Thai Baht, US $820). The questions inquiring about underlying medical conditions included hypertension, dyslipidemia, diabetes mellitus, chronic renal disease, visual impairments, and hearing impairments. We also gathered data on other related variables, such as wearing glasses or contact lenses, using hearing aids, and a number of current medications.

#### Experience Using Mobile Apps and mHealth

mHealth apps, or mobile health apps, refer to the practice of medicine and public health supported by mobile devices. The term “mHealth apps” encompasses the use of mobile communication devices such as smartphones and tablets for health care purposes [12,13]. For information on owning and experience using the devices and mHealth, questionnaires asked for smartphone or tablet usage experience (year), owning smartphones or tablets, internet usage experience (year), and previously used mobile apps and mHealth apps.

### mHealth Acceptance and ITU

The acceptance of mHealth was evaluated using the Thai mHealth Senior Technology Acceptance Model (STAM) questionnaires. This instrument has been adapted from the 38-item STAM questionnaire [14] and validated for accessing mHealth acceptance in a Thai context. According to the reported psychometric analysis, the Thai mHealth STAM demonstrated satisfactory psychometric properties in terms of validity and reliability. We used instrument items in the following domains as the potential associated factors of an ITU mHealth: attitude toward using, perceived usefulness, perceived ease of use, perceived barriers, gerontechnology anxiety, and facilitating condition. The structure of the Thai mHealth STAM was a 10-point Likert scale. The highest point indicated a high level of acceptance. The participant’s ITU mHealth was assessed by the structural question “If there are available mHealth apps for you, do you want to use them? (Yes/No).”

### Sample Size

To derive statistics that represent the parameters for the target populations, a minimum sample size of 500 total participants is required. This minimum sample size can accurately detect low to large effect sizes, as suggested by Bujang et al [15]. Based on our primary objective, it aimed to explore the associations between ITU and predetermined variables, including characteristics, ownership, and experience using devices and mHealth, and mHealth acceptance, in 2 specified subgroups of participants: those with experience using mHealth apps and those without. To explore unbiased effect estimates, 18 independent variables were analyzed with adjustments for 5 confounding variables. The sample size calculation, based on the formula n=100+(10‐50 event per variable)*i*, where *i* refers to the number of independent variables, was conducted to ensure our effect estimates from a multivariable logistic regression had sufficient precision to yield medium to large effect estimates in the subgroup analysis [15]. With 23 independent variables, a minimum sample size of 330 participants per subgroup was needed according to the given formula with 10 per independent variables.

### Statistical Analysis

For descriptive analysis, we presented categorical data using frequency and percentage. We described continuous data using a mean with SD, a median with a range (minimum-maximum), or an IQR, as appropriate. The comparison in characteristics, owning and experience using the devices and mHealth, and mHealth acceptance between participants who had ITU and those who did not was examined using an independent 2-tailed *t* test or Wilcoxon rank sum test for continuous data and Fisher exact test for categorical data.

For the analysis of the primary objective, we hypothesized that the factors associated with ITU would be different for participants who had previously used mHealth and those who did not. We performed a multivariable logistic regression for 2 specified subgroups, adjusted for the confounders including age, gender, education levels, income, and living area. The predetermined characteristic variables included having any underlying disease, owning a smartphone, the number of years of experience using it, and an interaction term based on both ownership and experience. We entered each item’s mHealth acceptance into the model as continuous data, using a 10-point scale. The magnitude of associations was presented using an adjusted odds ratio along with 95% CIs and *P *value. All statistical analyses were conducted using STATA (version 17.0; Stata Corp, LP). The visualization for this study was created using Microsoft Excel (Microsoft Corporation, 2021). The level of statistical significance for descriptive analysis was set at a *P* value below .05.

## Results

### Participant Characteristics

**Figure 1.**
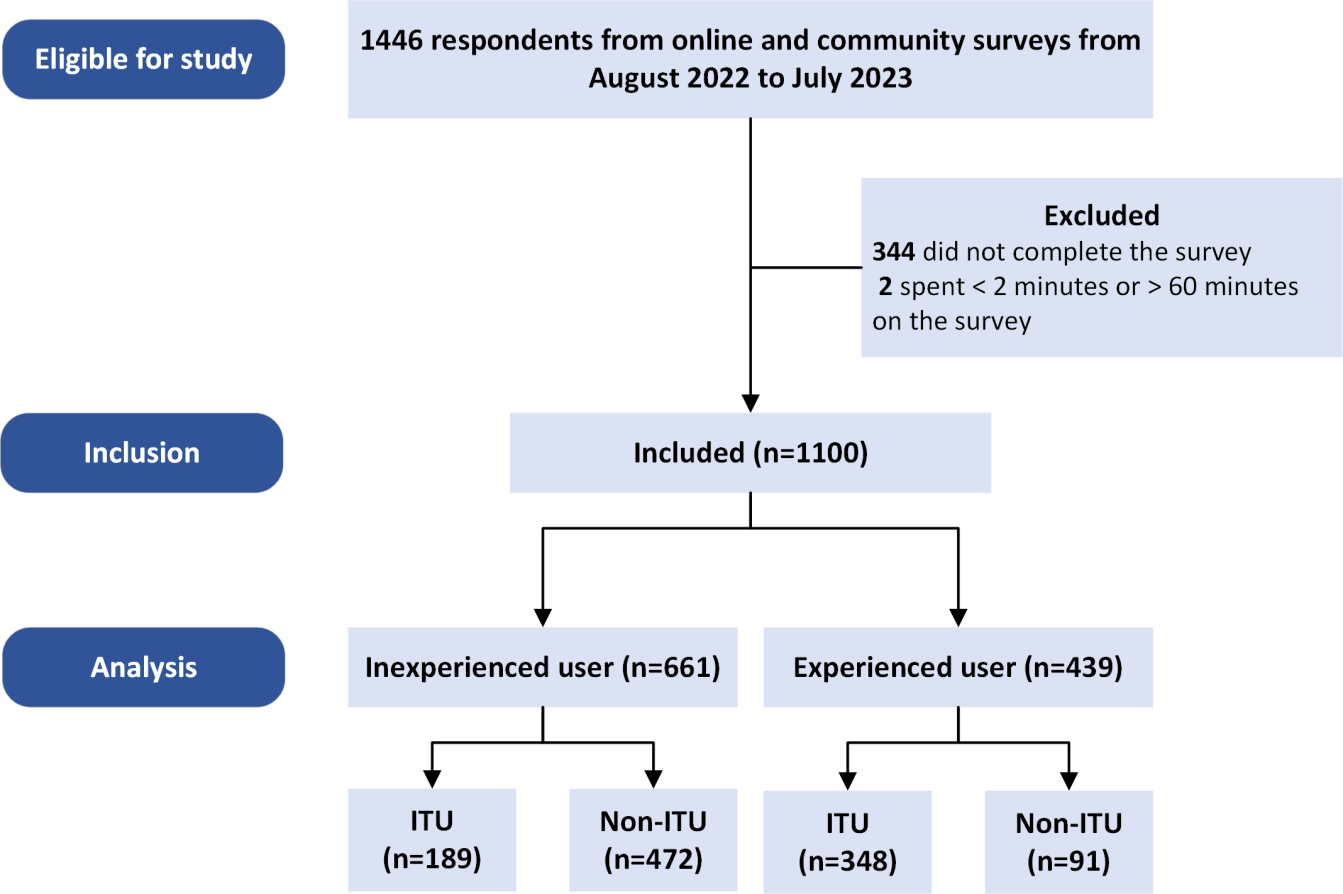
Study flow diagram. ITU: intention to use.

Of 1100 participants, the majority were female (776/1100, 62.3%), with a mean age of 62.3 (SD 8.8) years. This study flow diagram is presented in Figure 1. Most of the participants were married and resided with their families primarily in rural, suburban, and urban areas, respectively. For socioeconomic status, 86.2% (948/1100) had a low income (<10,000 Baht, US $274), and 65.9% (725/1100) had the highest education level at primary school. The common underlying diseases were hypertension and dyslipidemia. More than half reported having a vision problem, and 65.2% (n=399) wore glasses or contact lenses. Only 3.3% (4/1100) of respondents required a hearing aid, while 10.9% (120/1100) of respondents reported having hearing problems.

There were 537 participants who had ITU (48.8%) and 563 participants who did not (51.2%). When comparing the participants who had ITU mHealth apps with those who did not, the ITU group had a significantly younger average age, higher education levels, higher income, and a lower proportion of underlying diseases. Furthermore, 94.4% (507/537) and 92% (494/537) of the ITU group reported having experience using a smartphone or tablet and the internet; 91.2% (490/537) and 3.4% (18/537) had owned a smartphone and tablet, respectively. These percentages in the ITU group were significantly higher than those in the non-ITU group. The overall median (IQR) year of experience using the devices was 5 (0‐10). When comparing the ITU to non-ITU groups, the median years of device usage experience in the ITU were significantly higher than non-ITU (median 6, IQR 3‐10 versus median 3, IQR 0‐9, *P*<.001). In addition, 66.9% of those in the ITU group had experience using mHealth apps, which was a significantly higher proportion than the non-ITU group of 17.4%. The details of participant characteristics are presented in Table 1.

**Table 1.** Participant characteristics.

			Had intention to use mHealth apps		
Characteristics		Total (N=1100)	Yes (n=537)	No (n=563)	*P* value
Age (year), mean (SD)		62.3 (8.8)	60 (8.4)	64.4 (8.6)	<.001
Male, n (%)		324 (29.5)	140 (26.1)	184 (32.7)	.02
**Marital status, n (%)**					.002
Single		96 (8.7)	57 (10.6)	39 (6.9)	
Married		747 (67.9)	377 (70.2)	370 (65.7)	
Separated, divorced, or widowed		257 (23.4)	103 (19.2)	154 (27.4)	
**Education levels, n (%)**					<.001
No education		18 (1.6)	2 (0.4)	16 (2.8)	
Primary school		725 (65.9)	291 (54.2)	434 (77.1)	
Secondary school		97 (8.8)	65 (12.1)	32 (5.7)	
High school and vocational training		162 (14.7)	110 (20.5)	52 (9.2)	
Preuniversity		11 (1)	8 (1.5)	3 (0.5)	
Bachelor’s degree		79 (7.2)	54 (10.1)	25 (4.4)	
Master’s degree		8 (0.7)	7 (1.3)	1 (0.2)	
**Income per month, n (%)**					.001
<10,000 Baht (US $274)		948 (86.2)	442 (82.3)	506 (89.9)	
10,001‐30,000 Baht (US $275‐819)		138 (12.5)	86 (16)	52 (9.2)	
>30,001 Baht (US $820)		14 (1.3)	9 (1.7)	5 (0.9)	
**Living status, n (%)**					.86
Alone		108 (9.8)	50 (9.3)	58 (10.3)	
With family		988 (89.8)	485 (90.3)	503 (89.3)	
With others		4 (0.4)	2 (0.4)	2 (0.4)	
**Living area, n (%)**					.38
Urban		220 (20)	99 (18.4)	121 (21.5)	
Suburban		377 (34.3)	192 (35.8)	185 (32.9)	
Rural		503 (45.7)	246 (45.8)	257 (45.6)	
Had any underlying disease, n (%)		726 (66)	330 (61.5)	396 (70.3)	.002
Hypertension, n (%)		495 (45)	212 (39.5)	283 (50.3)	<.001
Dyslipidemia, n (%)		375 (34.1)	187 (34.8)	188 (33.4)	.62
Diabetes mellitus, n (%)		184 (16.7)	76 (14.2)	108 (19.2)	.03
Chronic kidney disease, n (%)		17 (1.5)	8 (1.5)	9 (1.6)	.88
Vision problems, n (%)		612 (55.6)	304 (56.6)	308 (54.7)	.53
Wore glasses or contact lens, n (%)		399 (65.2)	210 (69.1)	189 (61.4)	.045
Hearing problems, n (%)		120 (10.9)	54 (10.1)	66 (11.7)	.38
Used hearing aids, n (%)		4 (3.3)	2 (3.7)	2 (3)	.84
Number of medications, median (IQR)		1 (0‐2)	1 (0‐2)	1 (0‐2)	.55
Had experience using a smartphone or tablet, n (%)		873 (79.4)	507 (94.4)	366 (65)	<.001
Had own smartphone, n (%)		843 (76.6)	490 (91.2)	353 (62.7)	<.001
Had own tablet, n (%)		20 (1.8)	18 (3.4)	2 (0.4)	<.001
Had experience using the internet, n (%)		784 (71.3)	494 (92)	290 (51.5)	<.001
Had experience using mHealth apps, n (%)		439 (39.9)	348 (64.8)	91 (16.2)	<.001

### Experience Using Mobile Apps and mHealth

**Figure 2.**
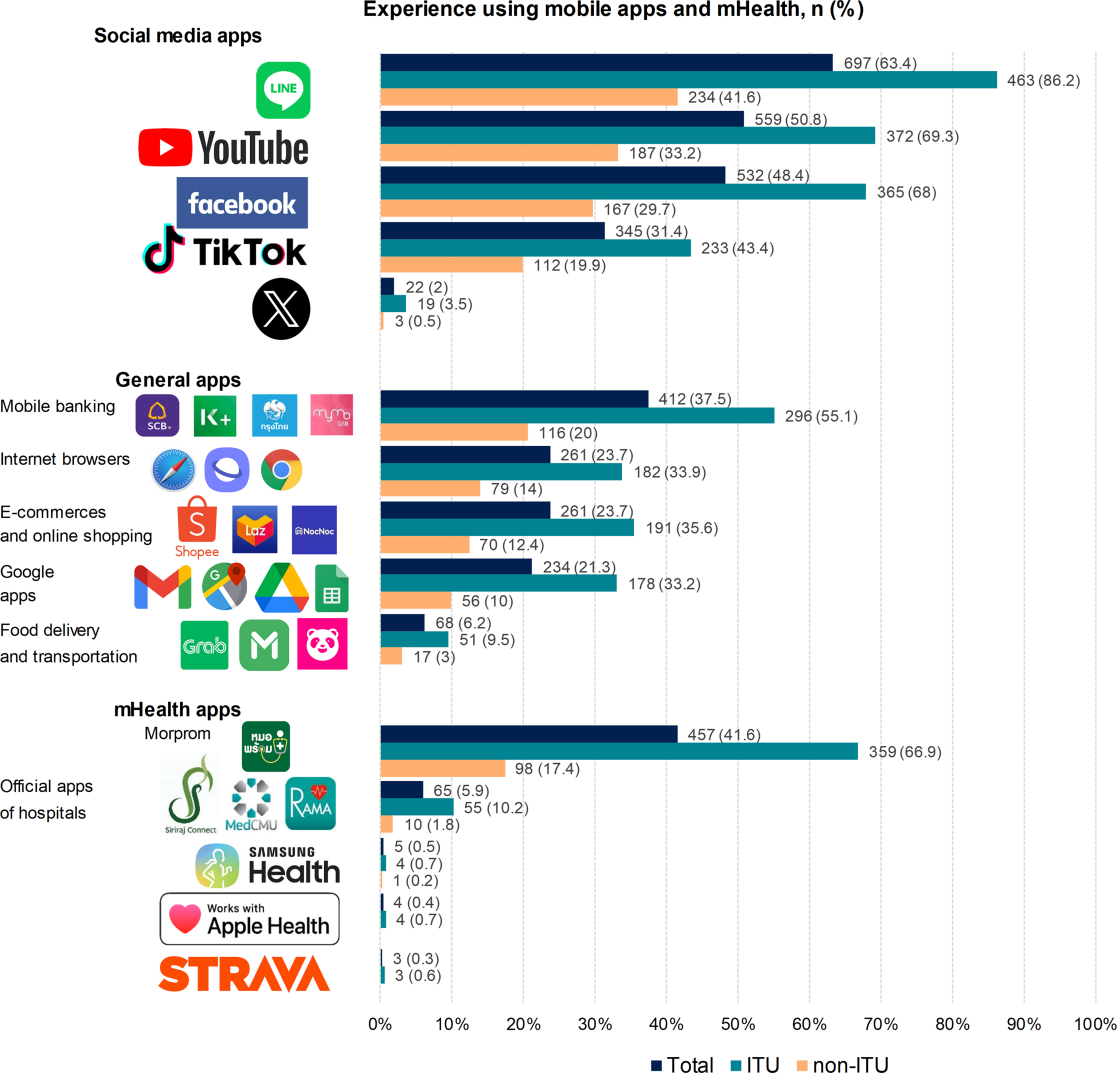
Participants’ experience using mobile apps and mHealth. ITU: intention to use.

Figure 2 illustrates participants’ experience using mobile apps and mHealth. There are many types of mobile apps and mHealth including social media apps, general apps, and mHealth apps. Among the middle-aged and older adults who had previously used mobile apps and mHealth, social media apps had the highest percentages (63.4%), followed by mHealth apps (41.6%), and apps for general purposes (37.5%). The use percentages of almost all mobile apps and mHealth were statistically and significantly higher in the ITU compared to non-ITU groups (*P*<.001), except for mHealth apps with a low use percentage (Samsung Health, Apple Health, and Strava). The use percentages of the top 3 social media apps (Line, YouTube, and Facebook) were greater than other apps. For general purposes, most participants used mobile apps for online banking services, surfing the internet, and shopping. For mHealth, the Ministry of Thai Public Health app, “MorProm,” had the highest use (41.6%), followed by the official apps by hospitals (5.9%), while the other mHealth apps from public providers showed a very low use percentage.

### mHealth Acceptance

**Figure 3.**
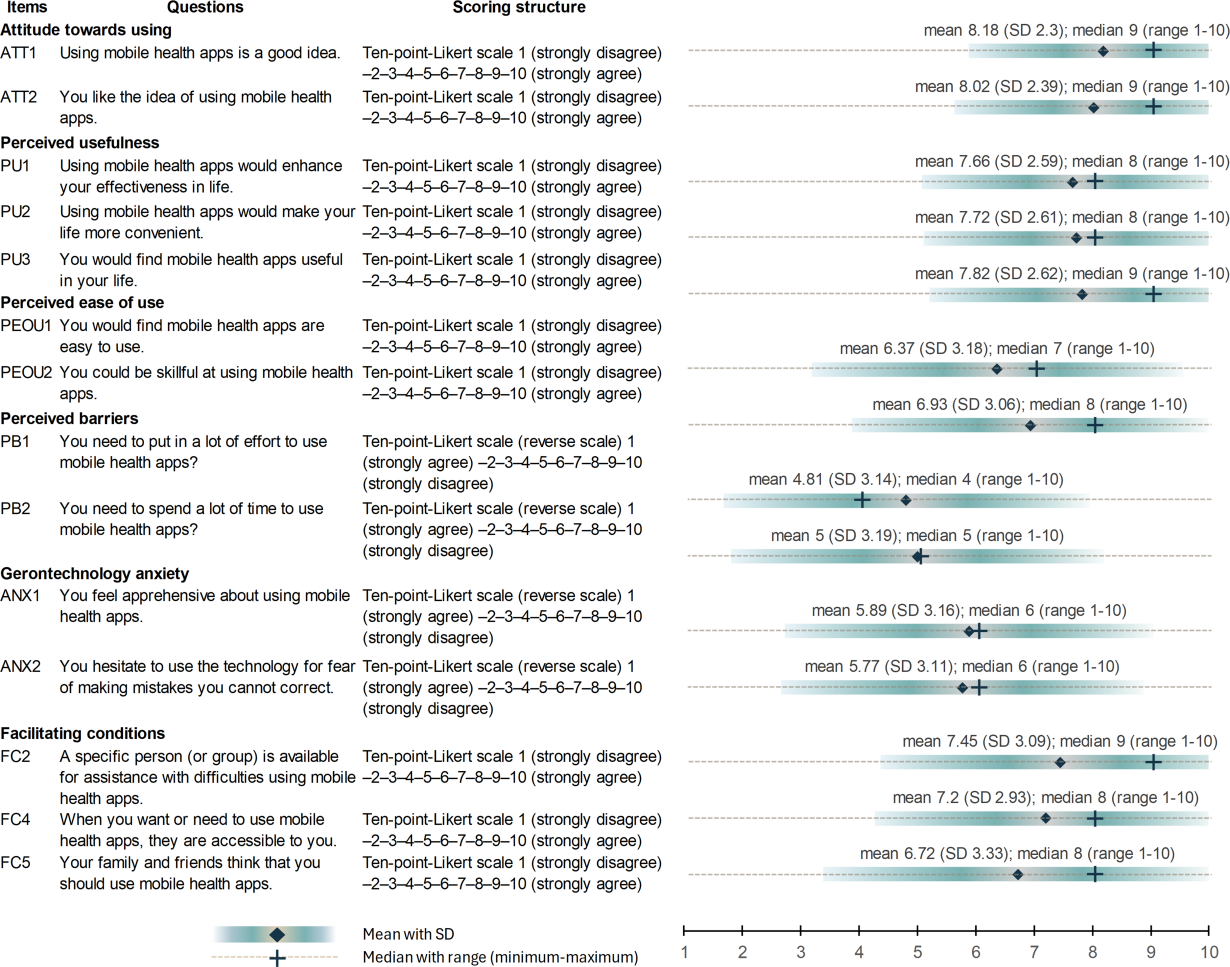
Participants’ responses to mHealth acceptance questionnaires. ANX: gerontechnology anxiety; ATT: attitude toward using; FC: facilitating condition; PB: perceived barriers; PEOU: perceived ease of use; PU: perceived usefulness.

We assessed the participants’ mHealth acceptance using the Thai mHealth STAM questionnaires in the domains, including attitude toward using, perceived usefulness, perceived ease of use, perceived barriers, gerontechnology anxiety, and facilitating condition. Figure 3 displays the structure of the questionnaires and the participants’ responses. Overall, participants’ acceptance of mHealth fell within the range of agreeable attitudes toward using, perceived usefulness of mHealth apps, and facilitating conditions. On the other hand, we observed responses in the range of neutral to low acceptance across the domains of perceived barriers, gerontechnology anxiety, and perceived ease of use.

When compared between the ITU and non-ITU groups, mHealth acceptance levels assessed by the questionnaires showed a significant difference in all items, as presented in Table S1 in Multimedia Appendix 1. Participants who used an ITU mHealth app had higher mean scores in all items. The domain of perceived barriers yielded the lowest mean mHealth acceptance score in both groups.

### Factors Associated With ITU mHealth Apps

**Figure 4.**
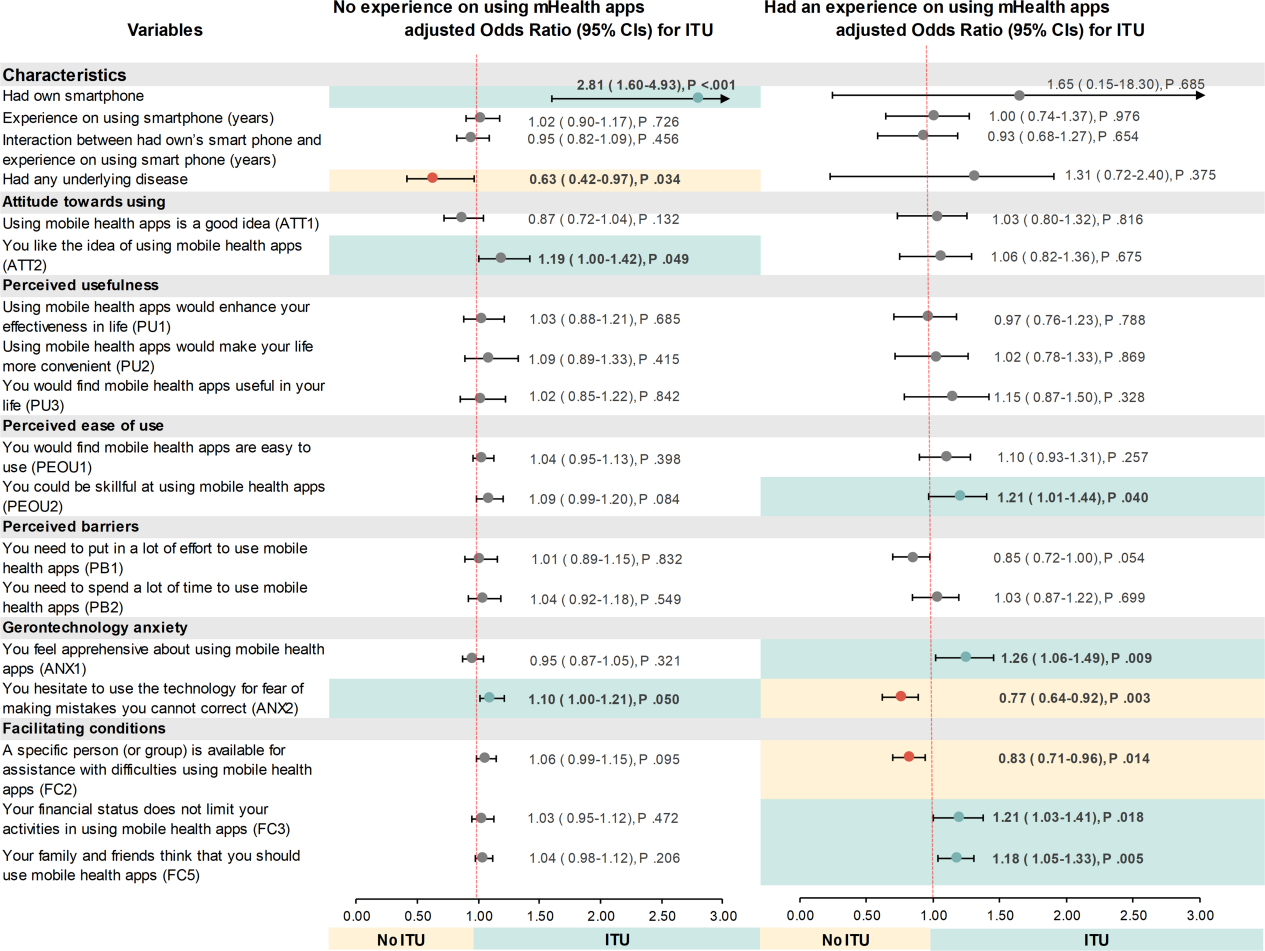
Factors associated with intention to use mHealth apps using a multivariable logistic regression. aORs were estimated as adjusted for age, gender, education levels, income, and living area. aOR: adjusted odds ratio; ANX: gerontechnology anxiety; ATT: attitude toward using; FC: facilitating condition; ITU: intention to use; PB: perceived barriers; PEOU: perceived ease of use; PU:perceived usefulness.

In this study, we focused on exploring factors associated with ITU mHealth apps in the subgroup of participants who had experience using mHealth apps and who did not. We performed a multivariable logistic regression analysis using predetermined factors including owning a smartphone, experience using a smartphone, underlying disease, and mHeath acceptances. The analysis also adjusted for the confounders consisting of age, gender, education levels, income, and living area. Multivariable analysis results are presented in Figure 4.

For the subgroup of people who had never used mHealth apps before, having a smartphone was substantially associated with higher odds of ITU (adjusted odds ratio 2.81, 95% CI 1.60 to 4.93; *P*<.001), while having any underlying disease was significantly related to a low ITU. Higher levels of acceptance, which are characterized by a higher score of attitudes toward the idea of using mHealth and a lower fear of making mistakes that cannot be corrected when using it, were also significantly associated with a high ITU.

For the subgroup of individuals who had prior experience using mHealth apps, the pattern of associations between predetermined factors varied from the subgroup of those who did not have prior experience. There were no predetermined characteristics relating to ITU. The levels of mHealth acceptance were significantly associated with ITU in the domains, including perceived ease of use, gerontechology anxiety, and facilitating conditions. Perceived ease of use, which individuals could be skilled at using, was significantly associated with a greater odds of ITU. Regarding the level of anxiety associated with mHealth usage, less apprehensiveness about using mHealth significantly increased the odds of ITU. Conversely, a low ITU was associated with a lower fear of making mistakes when using mHealth. Having facilitated support from family and friends to use mHealth and having fewer financial constraints on using mHealth showed a significant positive association with ITU, whereas the support from others was negatively associated with ITU.

## Discussion

### Principal Findings

To investigate the factors associated with ITU, we found that people who had never used mHealth apps before, having a smartphone associated with a higher ITU, while having any underlying disease was significantly related to a low ITU. Higher levels of acceptance, which are characterized by a higher score of attitudes toward the idea of using mHealth and a lower fear of making mistakes, were also significantly associated with a high ITU. For the subgroup of individuals who had prior experience using mHealth apps, there were no predetermined characteristics relating to ITU. The levels of mHealth acceptance were significantly associated with ITU in the domains, including perceived ease of use, gerontechology anxiety, and facilitating conditions.

Compared to previous studies, several patient characteristics significantly influence the ITU mHealth. Older patients are less likely to use mHealth due to their unfamiliarity with technology. Despite efforts to adopt new technology, many older adult individuals still struggle with it, and the rapid pace of technological advancements leaves them unable to keep up [16,17]. Additionally, there is a lack of promotion and support for using medical technology among older adults [18]. Socioeconomic status is another crucial factor affecting the ITU mHealth [19,20]. Patients in low-income groups not only lack support for using mHealth, but their daily routines and various burdens also reduce their intention to use these technologies [21]. This is closely related to the level of education [19]; patients with higher educational levels are more likely to understand and recognize the health benefits of mHealth, thus increasing their intention to use it [20]. Chronic diseases also play a role [19]. Patients without comorbidities and those who could effectively manage their chronic conditions tend to have better health management skills and a stronger desire to maintain good health, which positively influences their ITU mHealth [22,23].

Many factors influence the ITU mHealth among people without prior experience. One critical factor is the availability of equipment, such as mobile phones, patient testing devices, personal digital assistants, and other wireless tools. Health care professionals must ensure these facilities are accessible to patients when initiating mHealth services. Providing the necessary equipment and educating patients on its use can reduce inequity, particularly among older populations. The previous studies also showed that when health care systems provide access to such equipment, it enhances user confidence and intention to adopt mHealth, particularly among older adults who may otherwise face barriers due to digital inequality (eg, lacking smartphones or internet access) [24,25]. Empowering patients to access and use smartphones not only enhanced their ITU mHealth but could also improve self-rated, physical, and psychological health levels, as evidenced by the previous studies on the effects of smartphones and smart devices [26-28]. Underlying health conditions also impact the ITU mHealth. Patients with poor health behaviors or low motivation for self-care are less likely to adopt mHealth. The previous studies found that older adults with heart failure [29] and adults with hypertension [30] faced significant barriers to mHealth adoption, including low motivation and limited health literacy, which hindered their engagement with self-management technologies. These support our finding that underlying health conditions may reduce mHealth adoption, as individuals often lack the motivation to use health-promoting technologies. Conversely, some studies suggest that the presence of chronic health conditions can actually motivate individuals to engage with mHealth solutions. The study by Askari et al [31] found that older adults who were able to control their chronic diseases often expressed a greater ITU mHealth, as these tools can provide essential support for self-management and health monitoring. This indicated that while health conditions could pose barriers, they can also motivate the adoption of mHealth solutions, particularly when patients recognize the potential benefits for their health management. Another study also suggested that health conditions could influence older adults’ readiness to engage with eHealth resources, with those experiencing chronic illnesses showing a higher willingness to use digital health tools for self-care [32]. This suggests that the context of the health condition is vital in influencing attitudes toward technology use, rather than the notion that poor health behaviors uniformly result in reduced intentions to use mHealth. Confidence in their ability to effectively use mHealth apps is crucial, as a positive attitude toward mHealth increases the likelihood of its use [33]. The positive finding on attitude toward using mHealth is also consistent with the recent systematic reviews on the impact of mHealth interventions, which highlighted that the effectiveness of these interventions is often linked to user confidence and positive attitudes toward technology [4]. To promote mHealth for people without prior experience, health care professionals should educate patients about its benefits and how it can aid in self-care. Additionally, patients must develop eHealth literacy, defined as “the ability to seek out, find, evaluate, and appraise, integrate, and apply what is gained in electronic environments toward solving a health problem.” [34].

For participants with prior experience using mHealth, various factors influence their intention to continue its usage. These individuals often possess a positive attitude toward mHealth and recognize its benefits. They may also have experience in overcoming barriers, such as troubleshooting difficulties or correcting errors independently. Consequently, they perceive the benefits of using mHealth to outweigh the risks or challenges. Our finding indicated that perceived self-capability to use mHealth and lower concerns was associated with higher ITU among participants with prior mHealth experience. This finding is aligned with the study by Opoku et al [35], which emphasized that enabling resources, such as the durability and simplicity of mobile technology, significantly influence patients’ perceived ease of use of mHealth interventions. This implies that when patients are informed and experienced about the user-friendly nature of mHealth apps, their confidence in using these tools increases, thereby enhancing their likelihood of adoption. In contrast to participants without prior experience using mHealth, those with experience did not avoid using mHealth out of fear of making mistakes. However, the expressed greater concerns about mHealth itself, such as its effectiveness, data security, and privacy, were associated with a low ITU. Our findings align with the studies on experienced users’ attitudes toward mHealth [36,37], emphasizing the importance of addressing concerns related to security, privacy, and perceived effectiveness. Therefore, future mHealth interventions should not only focus on enhancing user confidence through health education or instruction by technical and health care providers but also on building trust in technology itself. Additionally, patients, especially older adults, value support from family or caregivers. mHealth for older adults may require assistance at various stages, including tool usage guidance, instructions, and caregiver involvement [16]. Financial considerations also play a crucial role, as increased costs associated with mHealth can affect the ITU for it. To initiate and improve mHealth usage, health care professionals need to identify patients willing to use mHealth, ensure the availability of necessary equipment, and address any financial constraints. Educating patients on the evidence and benefits of mHealth is also essential. Where possible, families and caregivers should be included in the mHealth care plan [38].

The strength of this study lies in its relatively large sample size, which included participants from rural, suburban, and urban areas. This study comprehensively explored various factors potentially associated with mHealth use, using reliable and validated standard tools to evaluate acceptance. However, some limitations existed. The cross-sectional nature of this study limited establishing causal relationships between the ITU mHealth and associated factors. Further research designs, including longitudinal studies or randomized controlled trials of specific mHealth interventions, are necessary to overcome these limitations and provide a more comprehensive understanding of the factors influencing mHealth usage. Furthermore, the term “mHealth” was defined in a broad and general sense in the questionnaire but was not explicitly specified for participants’ conditions. Defining “mHealth” in general could hinder the participants’ acceptance and use of mHealth, particularly in the group of participants with underlying diseases and specific health problems. This hypothesis could explain the observed association between having any underlying disease and low ITU in the group of participants who had never used mHealth before. Accordingly, our study primarily aimed to explore mHealth usage in general for community-dwelling adults. Our findings may not generalize enough to provide specific insight into the specific use case of mHealth in a particular population. Hence, we encourage future studies to aim for an in-depth understanding of each type of mHealth and acceptance in the specific context.

### Conclusion

This study identified key factors influencing the ITU mHealth and positive attitudes toward using significantly enhancing ITU in both inexperienced and experienced users. Conversely, having an underlying disease was associated with a decrease in ITU, indicating that this group of individuals may require mHealth’s specific requirements for their conditions rather than the general purpose. For experienced users, acceptance levels in areas such as ease of use and gerontechnology anxiety were critical. Health care professionals should identify patients open to using mHealth, ensure access to necessary equipment, address financial barriers, and educate patients on its benefits. Future investigations should also explore specific mHealth apps to provide more targeted insights.

## Supplementary material

10.2196/63607 Multimedia Appendix 1 Additonal table. The comparison of participants’ responses to mHealth acceptance questionnaires.

## Abbreviations

ITU: intention to use
mHealth: mobile health care
STAM: Senior Technology Acceptance Model
STROBE: Strengthening the Reporting of Observational Studies in Epidemiology
