# Supplementary material for: Factors Associated With the Intention to Use mHealth Among Thai Middle-Aged Adults and Older Adults: Cross-Sectional Study
Source: JMIR Hum Factors. 2025 Mar 7;12:e63607. doi: 10.2196/63607 (PMC11908770; doi:10.2196/63607)
Supplement: Multimedia Appendix 1 [file humanfactors-v12-e63607-s001.docx]

**Table S1.** The comparison of participants’ responses to mHealth acceptance questionnaires

| **Items** | **Questions** | **Total** | **Had intention to use mHealth apps** | | ***P* value** |  |
| --- | --- | --- | --- | --- | --- | --- |
|  |  |  | **Yes** | **No** |  |  |
|  |  | **N=1,100** | **n=563** | **n=537** |  |  |
| **Attitude towards using** | |  |  |  |  |  |
| ATT1 | Using mobile health apps is a good idea. | 8.2 (±2.3) | 9.0 (±1.6) | 7.5 (±2.6) | <0.001 |  |
| ATT2 | You like the idea of using mobile health apps. | 8.1 (±2.4) | 8.9 (±1.7) | 7.3 (±2.7) | <0.001 |  |
| **Perceived usefulness** | |  |  |  |  |  |
| PU1 | Using mobile health applications would enhance your effectiveness in life. | 7.7 (±2.6) | 8.6 (±1.9) | 6.8 (±2.9) | <0.001 |  |
| PU2 | Using mobile health apps would make your life more convenient. | 7.7 (±2.6) | 8.7 (±1.8) | 6.8 (±2.9) | <0.001 |  |
| PU3 | You would find mobile health apps useful in your life. | 7.8 (±2.6) | 8.9 (±1.8) | 6.9 (±2.9) | <0.001 |  |
| **Perceived ease of use** | |  |  |  |  |  |
| PEOU1 | You would find mobile health apps are easy to use. | 6.4 (±3.2) | 7.8 (±2.5) | 5.0 (±3.2) | <0.001 |  |
| PEOU2 | You could be skillful at using mobile health apps. | 6.9 (±3.1) | 8.4 (±2.1) | 5.5 (±3.2) | <0.001 |  |
| **Perceived barriers** | |  |  |  |  |  |
| PB1 | You need to put in a lot of effort to use mobile health apps? | 4.8 (±3.1) | 5.5 (±3.2) | 4.1 (±2.9) | <0.001 |  |
| PB2 | You need to spend a lot of time to use mobile health apps? | 5.0 (±3.2) | 5.8 (±3.2) | 4.2 (±3.0) | <0.001 |  |
| **Gerontechnology anxiety** | |  |  |  |  |  |
| ANX1 | You feel apprehensive about using mobile health apps. | 5.9 (±3.2) | 6.6 (±3.1) | 5.2 (±3.0) | <0.001 |  |
| ANX2 | You hesitate to use the technology for fear of making mistakes you cannot correct. | 5.7 (±3.1) | 6.4 (±3.1) | 5.1 (±3.0) | <0.001 |  |
| **Facilitating conditions** | |  |  |  |  |  |
| FC2 | A specific person (or group) is available for assistance with difficulties using mobile health apps. | 7.4 (±3.1) | 8.4 (±2.5) | 6.5 (±3.3) | <0.001 |  |
| FC4 | When you want or need to use mobile health apps, they are accessible to you. | 7.2 (±2.9) | 8.5 (±2.1) | 6.0 (±3.1) | <0.001 |  |
| FC5 | Your family and friends think that you should use mobile health apps. | 6.7 (±3.4) | 8.0 (±2.8) | 5.5 (±3.3) | <0.001 |  |

*p*-value was obtained by independent *t*-test.
